# Upregulation of caveolin-1 and its colocalization with cytokine receptors contributes to beta cell apoptosis

**DOI:** 10.1038/s41598-019-53278-z

**Published:** 2019-11-14

**Authors:** Gong Deuk Bae, Eun-Young Park, Kyong Kim, Se-Eun Jang, Hee-Sook Jun, Yoon Sin Oh

**Affiliations:** 1 Lee Gil Ya Cancer and Diabetes Institute, Department of Molecular Medicine, Gachon University, Incheon, South Korea; 2 College of Pharmacy, Mokpo National University, Jeonnam, South Korea; 3 Department of Food and Nutrition, Eulji University, Seongnam, South Korea; 4 College of Pharmacy and Gachon Institute of Pharmaceutical Science, Gachon University, Incheon, South Korea

**Keywords:** Cytokines, RNAi

## Abstract

Caveolin-1 (cav-1), the principal structural and signalling protein of caveolae, is implicated in various signalling events, including apoptotic cell death in type 2 diabetes. However, the precise role of beta cells in apoptosis has not been clearly defined. In this study, we investigated the involvement of cav-1 in cytokine-induced beta cell apoptosis and its underlying mechanisms in the rat beta cell line, INS-1 and isolated islets. Treatment of cytokine mixture (CM, TNFα + IL-1β) significantly increased the mRNA and protein expression of cav-1, and resulting in increased formation of caveolae. We found that IL-1 receptor 1 and TNF receptor localized to plasma membrane lipid rafts in the control cells and CM treatment recruited these receptors to the caveolae domain. After cav-1 siRNA transfection, CM-dependent NF-κB activation was reduced and consequently downregulated the mRNA expression of iNOS and IL-1β. Finally, decreased cell viability by CM treatment was ameliorated in both INS-1 cells and isolated islets treated with cav-1 siRNA. These results suggest that increased cav-1 expression and recruitment of cytokine receptors into caveolae contribute to CM-induced beta cell apoptosis.

## Introduction

Destruction of insulin-secreting pancreatic beta cells by autoreactive immune cells is one of the important causes of diabetes^1^. Interleukin-1β (IL-1β) and tumour necrosis factor-α (TNF-α) secreted by T cells and macrophages have been identified as the major mediators of beta cell failure during the development of diabetes^2,3^. Increased cytokines concentrations activate nuclear factor kappa-light-chain-enhancer of activated B cells (NF-κB) signalling pathway through integral receptors, IL-1 receptor 1 (IL-1R1) and TNF receptor (TNFR). Activation of IκB kinase (IKK) phosphorylates the IκBα protein, which results in ubiquitination and dissociation of IκBα from NF-κB. The activated NF-κB p65 is then translocated into the nucleus to promote expression of inflammatory genes and repress expression of genes involved in beta cell function^4–6^. These pathways are mediated by cytokine receptors in the plasma membrane, but little is known about how molecular components of the membrane regulate apoptotic pathways.

Caveolin-1 (Cav-1) is a member of the family of cholesterol binding membrane proteins that coat the intracellular surface of caveolae, which are small flask-shaped invaginations (50–100 nm in diameter)^7,8^. Cav-1 interacts with many caveolae-localized signalling molecules including G proteins, Src family tyrosine kinases, endothelial nitric oxide synthase (eNOS), and a number of ion channels via scaffolding domains within its NH_2_-terminal regions^9,10^. Therefore, cav-1 is a significant regulator of cellular signalling such as adhesion, apoptosis, migration, and aging process^11,12^. Cav-1 has been previously implicated in the development of diabetes via its role in insulin signalling and lipid metabolism in the liver, adipose tissues, and skeletal muscles^8^.

Recently, several studies about the role of cav-1 in pancreatic beta cells have been investigated. Nevins and Thurmond reported that inhibition of cav-1 in MIN-6 cells enhanced insulin secretion under physiological conditions (2.8 mM glucose)^13^. Moreover, in cells overexpressing cav-1, phosphorylation of cav-1 on tyrosine 14 promoted palmitate-induced cell death^14^ and internalization of insulin receptor^15^. However, the effect of cav-1/caveolae normally expressed in beta cells and their regulation in apoptosis under cytokine-induced toxicity remains unclear. Therefore, in this study, we investigated whether the involvement of cav-1 in cytokine induced beta cell apoptosis and its molecular mechanism in INS-1 cells and isolated islets.

## Result

### Cytokine mixture treatment increases cav-1 expression in INS-1 cells and isolated islets

**Figure 1:**
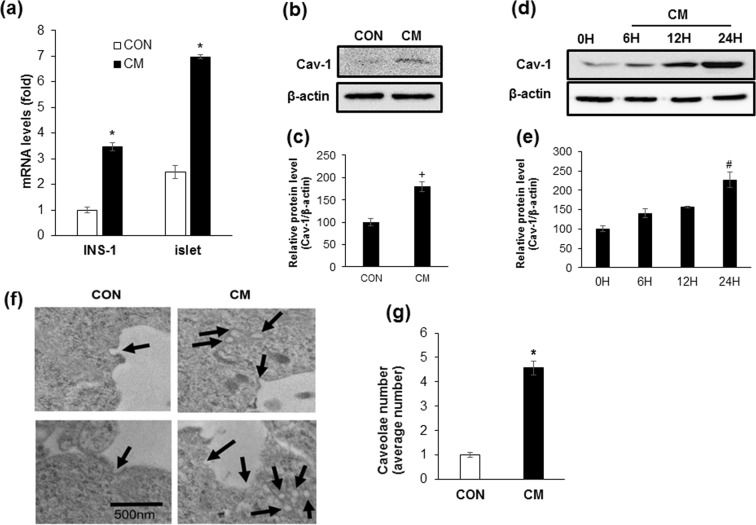
Cytokine mixture treatment increases cav-1 expression in INS-1 cells and isolated islets. (**a**) The mRNA level of cav-1 was determined after 6 h incubation with the cytokine mixture (CM; IL-1β 20 ng/ml, TNFα 20 ng/ml) in INS-1 cells and isolated islet from SD rat. (**b**) The amount of cav-1 protein was measured by western blot analysis after treatment of SD islet cells with CM for 24 h. (**d**) The amount of cav-1 protein was measured by western blot analysis after treatment of INS-1 cells with CM for various time periods (0 H, 6 H, 12 H, and 24 H). (**c**,**e**) The densities of western blot signals were measured, and the relative expression levels were normalized to that of actin. (**f**) Cells were treated with or without CM for 24 h and caveolae were observed by transmission electron microscopy. Bars: 500 nm. (**g**) Numeric counts of caveolae-like vesicles were statistically analysed in 10 independent cells. Values are means ± SEM from triplicate experiments, Arrows indicated caveolae structures. *p < 0.005 vs. CON, + p < 0.05 vs. CON, ^#^p < 0.005 vs 0 H.

To confirm whether cytokine mixture (CM, IL-1β + TNF-α) treatment induced beta cell apoptosis as previously^16,17^, INS-1 cells were treated with 20 ng/ml of IL-1β and TNF-α, and changes of cell viability was measured by the MTT assay and Annexin-V staining. Cell viability was significantly reduced by CM following both 24 and 48 h of treatment. Annexin-V stained cells (%) were also increased by 4.3-fold in CM treated cells compared with control (Supplementary Fig. 1a–c). We found that the expression levels of IL-1R1 and TNFR1 were significantly increased by CM treatment. Moreover, increased phosphorylation of IKKα, IKKβ, and IκBα in response to CM was observed. Lastly, nuclear translocation of the phosphorylated form of NF-κB p65 was significantly increased (Supplementary Fig. 1d,e). Next, to investigate the changes of cav-1 expression by cytokine toxicity in beta cells, INS-1 cells and isolated islets from SD rats were treated with CM for 6 h, and mRNA levels were analysed via qRT-PCR. As shown in Fig. 1a, mRNA expression of cav-1 in INS-1 cells or isolated islets treated with CM was significantly increased as compared with control (Fig. 1a). When we observed protein level in isolated islets and INS-1 cells, a significant increase was observed after 24 h of treatment compared with control (CON, 0 H) (Fig. 1b–e). Next, we examined caveolae structures by transmission electron microscopy (TEM) in control and CM-treated cells. As shown in Fig. 1f,g, the amount of caveolae in the cell membrane and cytosol was significantly increased by CM treatment (Fig. 1f,g). As cav-1 is known to phosphorylated on tyrosine-14 (pY14) in response to cytotoxic stress and sensitize to cell death^18,19^, we examined whether pY14-cav-1 was induced by CM treatment. As shown in Supplementary Figure 2, CM augmented cav-1 tyrosine-14 phosphorylation after 2 h treatment.

### Cytokine mixture treatment increases recruitment of cytokine receptors into caveolae in INS-1 cells

**Figure 2:**
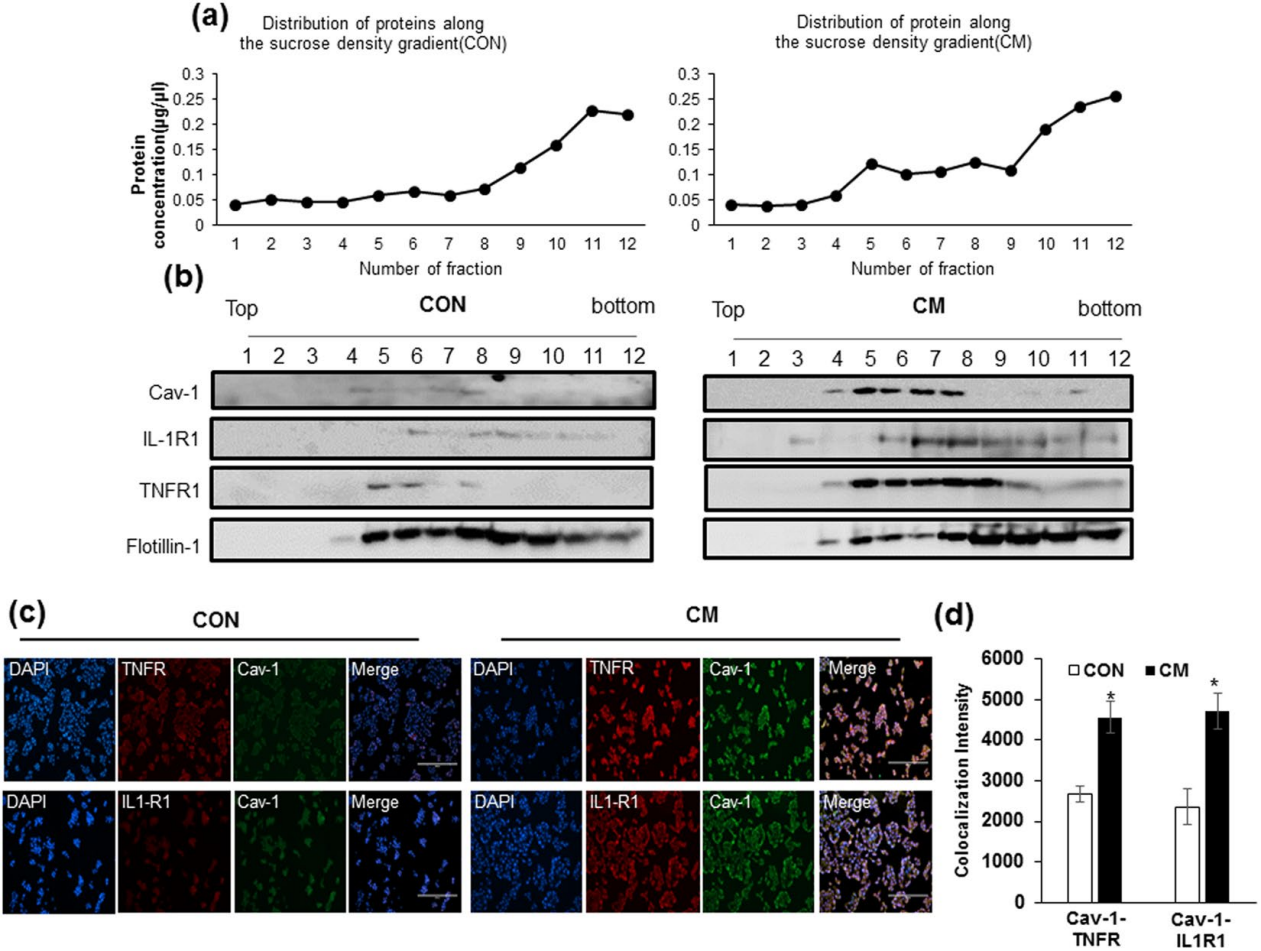
Cytokine mixture treatment increases recruitment of cytokine receptors into caveolae in INS-1 cells. INS-1 cells were incubated for 24 h with the cytokine mixture (CM; IL-1β 20 ng/ml, TNFα 20 ng/ml) and sucrose density gradient fractions were obtained from the cell lysates. Fractions are numbered 1 through 12 from top to bottom. (**a**) Protein levels in each fraction from CON and CM treated cells. (**b**) Westernblot with anti-cav-1, anti-IL-1β receptor (IL-1R1), anti-TNF receptor (TNFR), and anti-flotillin-1 antibodies. Flotillin-1 was used as a lipid raft marker. (**c**) The colocalization of IL-1R1, TNFR and cav-1 was confirmed by confocal microscopy. Nuclei were counterstained with DAPI (Blue). (**d**) Colocalization intensity was measured by Image J. Scale bars, 200 μm. Values are means ± SEM from triplicate experiments. *p < 0.05 vs. CON.

To investigate whether cav-1 affected the activation of NF-κB signalling, we analysed the localization of IL-1R1 and TNFR1 by CM stimulation. Control and CM treated cells were fractionated by sucrose gradient density centrifugation in the absence of detergent, a procedure widely used to isolated caveolae-enriched membrane domain^20^. Western blot analysis of the fractions showed that the majority of the cav-1 was present at the low density 5~30% sucrose interface of the gradient (Fig. 2b, Fractions 4–8). Protein amounts in each fraction showed that the CM treated cells contained higher levels of protein in the caveoale domain. (Fig. 2a). A significant proportion of IL-1R1 and TNFR1 was recruited to the caveolae fractions in CM treated cells, whereas flotillin-1, a membrane lipid raft marker, was similar in control and CM treated INS-1 cells. When control and CM-treated cells were co-immunostained with anti-IL1R1, anti-TNFR1 and anti-cav-1 antibodies, the receptors were found to be colocalized with cav-1 in the membrane of CM treated cells compared with control cells (Fig. 2c). Mean fluorescent intensity was calculated and showed a significant increase in IL-1R1 and TNFR1 localization with cav-1 protein in CM treated INS-1 cells compared with control (Fig. 2d).

### Down-regulation of cav-1 expression protects against cytokine mixture-induced beta cell apoptosis

**Figure 3:**
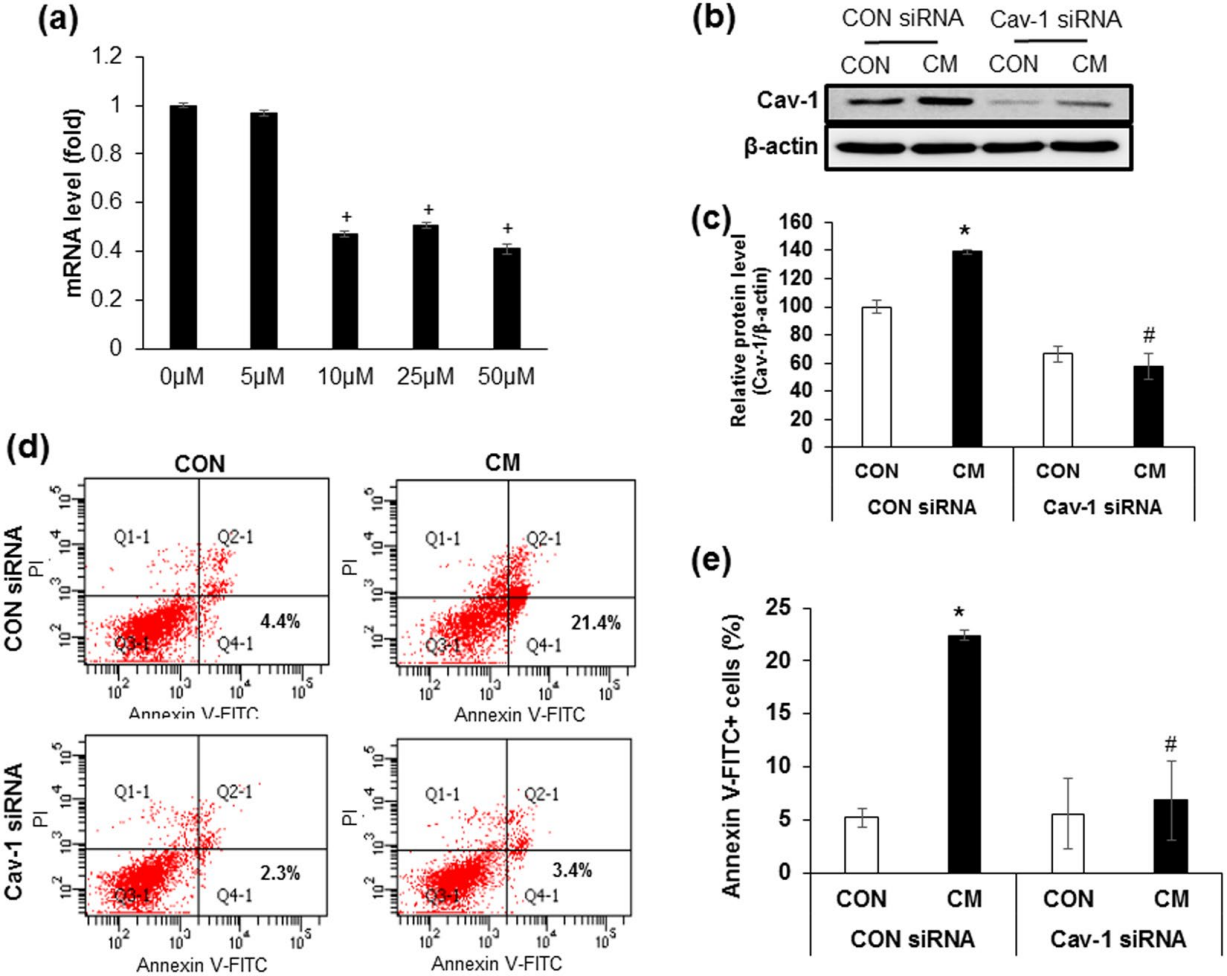
Down-regulation of cav-1 expression protects against cytokine mixture-induced beta cell apoptosis. (**a**) Cav-1 mRNA was measured after transfection with cav-1 siRNA at various concentrations (5, 10, 25, and 50 μM) for 24 h. (**b**) INS-1 cells were transfected CON siRNA (10 μM) or Cav-1 siRNA (10 μM) for 24 h. The amount of cav-1 protein was determined by western blot analysis after 24 h incubation with the cytokine mixture (CM; IL-1β 20 ng/ml, TNFα 20 ng/ml). (**c**) The densities of western blot signals were measured, and the relative expression levels were normalized to that of actin. (**d**) Cells were transfected with CON siRNA or Cav-1 siRNA for 24 h, followed by CM treatment. After 24 h, the cells were harvested and stained with Annexin V/propidium iodide, and apoptotic cells were evaluated by flow cytometry. (**e**) Quantitative data demonstrating the Annexin-V-FITC^+^ cells (%) (lower right quadrant). Values are means ± SEM from triplicate experiments, + p < 0.05 vs. 0 μM, *p < 0.05 vs. CON siRNA treated with CON, ^#^p < 0.05 vs. CON siRNA treated with CM.

**Figure 4:**
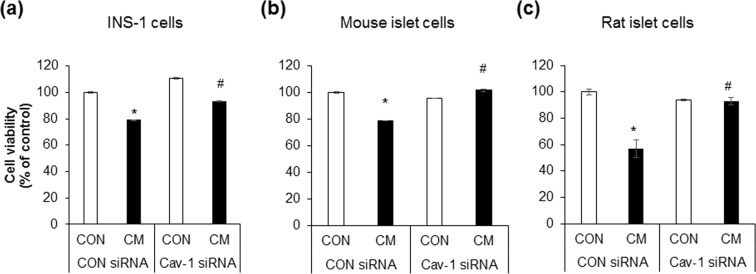
Down-regulation of cav-1 expression in INS-1 and isolated islets reduced cytokine mixture-induced cell damage. (**a**) CON siRNA (10 μM) or Cav-1 siRNA (10 μM) were transfected in INS-1 cells for 24 h. Cell viability was measured via the MTT assay after 24 h treatment with the cytokine mixture (CM; IL-1β 20 ng/ml, TNFα 20 ng/ml). The islets were cultured for 24 h following isolation from male C57BL/6 J mice (**b**) and male SD rat (**c**). Isolated islets were treated as described in (**a**) and cell viability was measured via the MTT assay. Values are means ± SEM from triplicate experiments, *p < 0.05 vs. CON siRNA treated with CON, ^#^p < 0.05 vs. CON siRNA treated with CM.

Given that recruitment of cytokine receptors into caveolae was increased by CM treatment, we investigated whether reduction of cav-1 expression could protect against beta cell apoptosis induced by CM. First, we downregulated cav-1 expression using 21-nucleotide siRNA at concentrations of 5, 10, 25, and 50 μM and found a marked reduction in cav-1 mRNA after 24 h of 10 μM siRNA transfection (Fig. 3a). We confirmed that protein expression of cav-1 was also reduced in cells treated with 10 μM of cav-1 siRNA, compared with control siRNA treated cells (Fig. 3b,c). To investigate whether decreased cav-1 expression would affect the levels of beta cell apoptosis, we transfected INS-1 cells with cav-1 siRNA in the presence or absence CM, and measured the level of apoptotic cells via annexin-V staining followed by flow cytometry analysis. As shown in Fig. 3d,e, the percentage of annexin-V-FITC positive cells increased during CM treatment, but was significantly reduced in cav-1 siRNA transfected cells (Fig. 3d,e). Moreover, cav-1 siRNA transfected cells had significantly higher cell viability upon CM treatment compared to control siRNA transfected cells (Fig. 4a). To confirm that downregulation of cav-1 affected CM-induced cell viability in islets, we transfected cav-1 siRNA into isolated islet cells from mice and rats. As shown in Fig. 4b,c, CM treatment significantly reduced cell viability compared to control cells treated with control siRNA and the reduction was attenuated by cav-1 siRNA in CM treated cells (Fig. 4b,c). In contrast, cells overexpressing cav-1 were more sensitive to CM treatment, and cell viability was dramatically decreased in cells transfected with pcDNA-cav-1 compared with cells transfected with control vector (Supplementary Fig. 3).

### Down-regulation of cav-1 expression inhibits cytokine mixture-induced NF-κB activation

**Figure 5:**
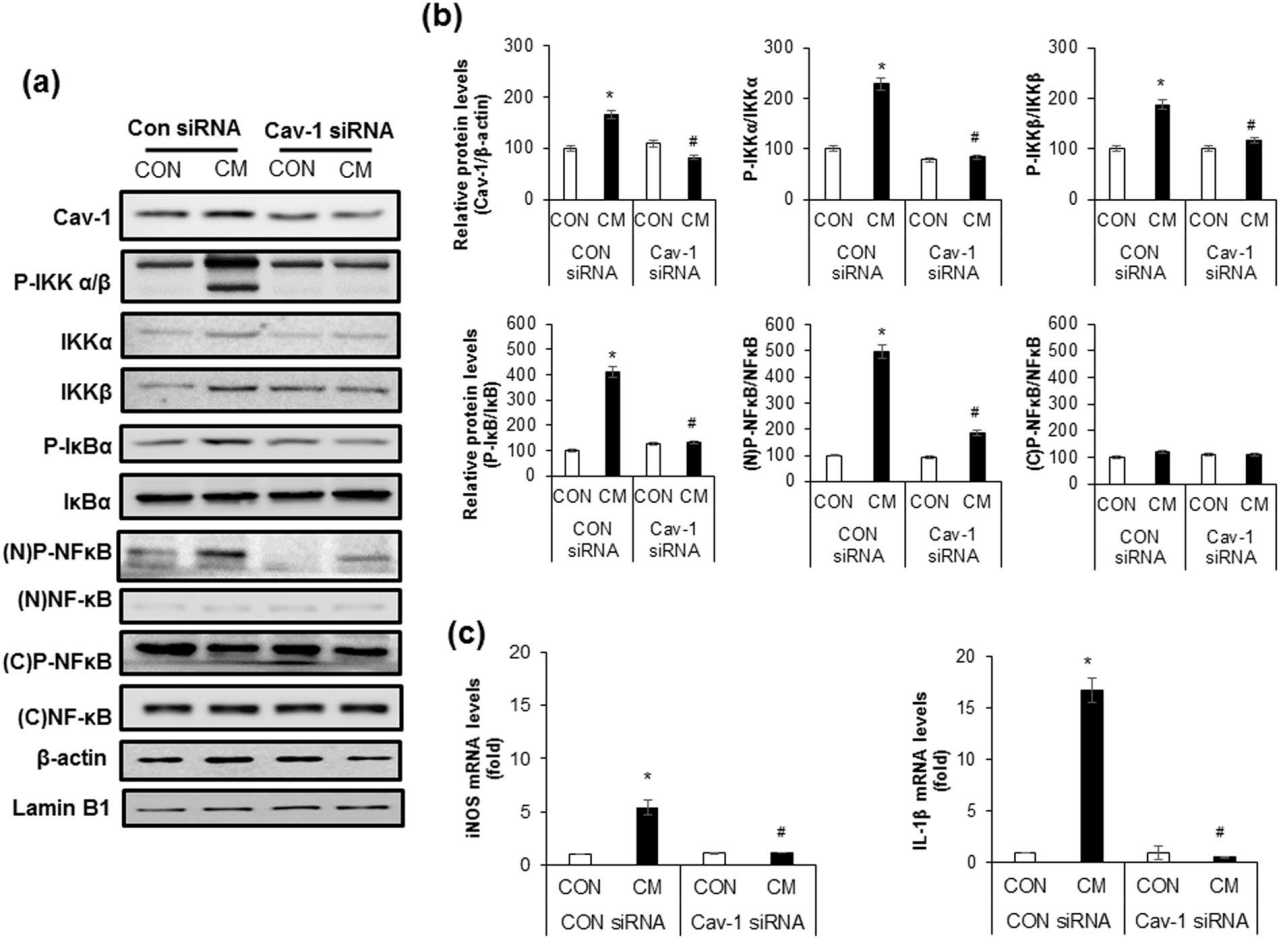
Down-regulation of cav-1 expression inhibits cytokine mixture-induced NF-κB activation. (**a**) INS-1 cells were transfected with CON siRNA or Cav-1 siRNA for 24 h, followed by treatment with the cytokine mixture (CM; IL-1β 20 ng/ml, TNFα 20 ng/ml). After 24 h, cell lysates were subjected to western blot analysis with specific antibodies. Actin and Lamin B1 were used as cytosolic and nuclear loading controls, respectively. (**b**) The densities of western blot signals were measured, and the relative expression level was normalized to that of actin, Lamin B1, and the non-phosphorylated form (IKK, IκB, and NF-κB). (**c**) Cells were treated as described in (**a**) and mRNA levels of IL-1β and iNOS was determined after 6 h incubation with the CM. Values are means ± SEM from triplicate experiments, *p < 0.05 vs. CON siRNA treated with CON, ^#^p < 0.05 vs. CON siRNA treated with CM.

To investigate whether CM-induced activation of NF-κB was affected by cav-1 expression, we analysed the expression levels of phosphorylated IKKα, IKKβ, and IκBα in cav-1 siRNA treated cells. As shown in Fig. 5a,b, the levels of phosphorylated IKKα, IKKβ, and IκBα by CM treatment were significantly reduced in cav-1 downregulated cells compared with control siRNA treated cells. Moreover, cav-1 downregulation significantly attenuated CM-induced NF-κB p65 phosphorylation in the nucleus (Fig. 5a,b). To investigate whether downstream target of NF-κB activation was affected by cav-1 depletion, mRNA expression level of IL-1β and inducible nitric oxide synthase (iNOS)^21^ was examined in cav-1 siRNA treated cells with or without CM. We found that mRNA expression of these genes were significantly increased after 6 h treatment of CM and the levels were reduced by cav-1 downregulated cells (Fig. 5c).

### Down-regulation of cav-1 expression increases glucose-stimulated insulin secretion

Since previous reports have shown that inhibition of cav-1 expression enhances insulin secretion^13^, we measured insulin secretion in the presence of 3 mM and 17 mM glucose after transfection with control and cav-1 siRNA in INS-1 cells. Insulin secretion in cells transfected with control siRNA was increased approximately 2.5-fold upon treatment with 17 mM glucose compared to treatment with 3 mM glucose. Moreover, transfection with cav-1 siRNA significantly increased insulin secretion upon treatment with both 3 mM and 17 mM glucose compared with control cells (Supplementary Fig. 4).

## Discussion

In this study, we elucidated the role of cav-1 in cytokine-induced beta cell apoptosis and also attempted to determine its underlying mechanisms in the INS-1 cell line. Prolonged exposure to pro-inflammatory cytokines, particularly the combination of IL-1β and TNFα, induced pancreatic beta cell apoptosis, which is one of the major causes of islet inflammation during the development of diabetes. Activation of NF-κB via phosphorylated IKKα/IKKβ and NF-κB p65 translocation into the nucleus is essential for the progressive loss of beta cells in cytokine-induced diabetes, and the inhibition of this process could be an effective strategy for beta cell protection^16,17,22,23^. As previously reported, there are many potential mechanisms of cytokine-induced beta cell death^1^, and receptor-mediated signalling strongly contributes to the activation of intracellular death signals, including NF-κB signalling^24^.

We found that expression levels of IL-1R1 and TNFR1 were increased by cytokine treatment. A study by Boni-Schnetzler *et al*. demonstrated that IL-1R1 expression is induced by palmitate and blocking IL-1R strongly inhibited proinflammatory factors stimulated by free fatty acids in human and mouse islets^25^. Moreover, IL-1R antagonist improves glycemia and beta cell secretory function in patients with type 2 diabetes^26^. TNF-induced cell death was also mediated by TNFR1^27^, and anti-TNF therapeutics are currently used to treat inflammatory diseases such as rheumatoid arthritis and Crohn’s disease^28^. These results suggest that increased expression and activation of these receptors may play an important role in beta cell inflammation during the development of diabetes.

Cav-1 plays an important role in metabolic signalling and is involved in glucose and/or cholesterol homeostasis, vesicle transport, proliferation, and apoptosis^12^. Catalan *et al*. demonstrated that expression of cav-1 mRNA is elevated in both visceral and subcutaneous adipose tissue of obese type 2 diabetic patients compared to lean controls^29^ and Wehinger *et al*. reported that overexpression of cav-1 in beta cells promoted apoptosis^14^. In the present study, CM treatment increased the number of caveolae structures, as well as the mRNA and protein expression levels of cav-1. These results suggest that upregulation of cav-1 plays an important role in transmitting signals from the cell surface via intracellular signalling pathways that regulate inflammation and type 2 diabetes.

Cav-1 is known to interact with many caveolae-localized signalling molecules via caveolin scaffolding domain (CSD) and an aromatic-rich caveolin binding motif (CBM) on the associated proteins^30^. Members of the TNF receptor subfamily contain a CBM motif and IL-1R1 effectors (IRAK, TRAF6, and IκB) are recruited to cav-1-containing lipid rafts^31,32^. The finding that cytokine signalling resulted in the recruitment of two receptors in caveolae on the plasma membrane is consistent with previous studies, and suggests that cav-1 dependent lipid rafts are critical for cytokine activation.

We found that inhibition of cav-1 reduced CM-induced NF-κB activation and lead to a decrease in mRNA expression of target genes (IL-1β and iNOS). Oakley *et al*. reported that IL-1R mediated NF-κB signalling was dependent on cav-1, as evidenced by the fact that inhibiting lipid raft formation reduced IL-1β-mediated NF-κB activation^31^. Garrean *et al*. also observed a significant reduction in LPS-induced IκBα degradation and NF-κB activation in Cav-1^−/−^ lungs relative to wild type mice^33^. These findings, in addition to those reported here, support a role for cav-1 in NF-κB activation in beta cells.

Recently, it was demonstrated that inhibition of cav-1 expression protected beta cell death induced by palmitate. Wehinger *et al*. found that over expression of cav-1 in MIN-6 cells promoted palmitate-induced beta cell apoptosis and implicated Src family kinase-mediated tyrosine phosphorylation in this pathway^14^. Zeng W *et al*. also reported that cav-1 depletion decreased lipotoxicity and increased proliferation via reduction of palmitate-mediated endoplasmic reticulum stress and cell cycle inhibitor expression^34^. Serio *et al*. demonstrated that phosphorylation of cav-1 on tyrosine 14 was observed in MIN-6 (mouse beta cells) cells overexpressing cav-1 upon palmitate treatment^14^, and this phosphorylation is associated with enhanced sensitivity in response to various cytotoxic stimuli^18,35^. In this study, we confirmed that CM also phosphorylated tyrosine-14 of cav-1, and our results suggested that cav-1 phosphorylation on tyrosine-14 augmented sensitivity to CM in INS-1 cells, restoration of CM induced beta-cell apoptosis might be also caused by loss of cav-1 phosphorylation site tyrosine-14.

It was reported that cav-1 depletion *in vitro* and *in vivo* results in insulin secretion. When unstimulated condition (low glucose level), cav-1 bound to insulin granule proteins including cdc42, guanosine 5′-triphosphate and vesicle associated membrane protein 2, but stimulation with glucose induced the dissociation of cav-1 from insulin granules and promoted insulin secretion^13^. Additionally, cav-1-deficient mice had higher plasma insulin levels and postprandial hyperinsulinemia under fasting or high-fat diet conditions^11^. Moreover, Wen *et al*. also demonstrated that cav-1 silencing significantly enhanced insulin production and secretion^34^. Therefore, these results demonstrate that downregulation of cav-1 could protect beta cells from CM treatment via reduced apoptosis and induced insulin secretion.

Previous study demonstrated that cav-1 deficiency mice showed lean phenotype and developed insulin resistance^11^, suggesting that the plasma insulin levels are affected by insulin action in peripheral tissues as well as insulin secretion in beta-cells. Although cav-1 deficiency in INS-1 cells results in increase insulin secretion compared with control cells under physiological glucose concentration, but whether hypoglycemia occurs and physiological role of cav-1 in insulin secretion in *in vivo* will be investigated in beta cell specific cav-1 KO mice.

**Figure 6:**
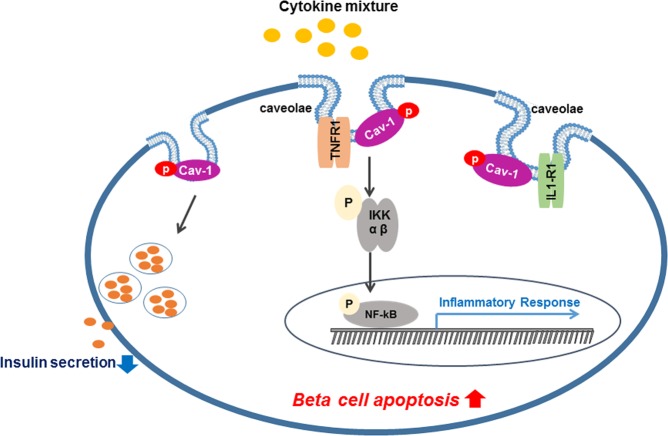
Schematic of the mechanism by which involvement of cav-1 and caveolae in CM-induced beta cell apoptosis in pancreatic beta-cells. Cytokine mixture treatment into beta cells inhibited insulin secretion and induced apoptosis. Cytokine mixture treatment increased caveolae structure as well as cav-1 expression and cytokine receptors (TNFR1 and IL1-R1) were recruited into caveolae. Therefore, activation of NF-kB signaling pathway increased the expression level of inflammatory response genes, which leads to beta cell apoptosis.

In summary, we proposed a schematic mechanism (Fig. 6) in which cav-1 is involved CM-mediated beta cell apoptosis. Increased expression of cav-1 and caveolae structure was observed in CM-treated cells and recruitment of cytokine receptors into caveolae contributed to CM-induced beta cell apoptosis. Moreover, silencing cav-1 expression inhibited CM-mediated NF-κB activation and increased insulin secretion, as well as cell viability. These results suggest that cav-1 as a potential target molecule in beta cell inflammation via the attenuation of CM induced beta cell apoptosis.

## Methods

### Cell culture

INS-1 rat insulinoma cells were grown in RPMI 1640 medium (Thermo Fisher Scientific, MA, USA) supplemented with 10% foetal bovine serum (Thermo Fisher Scientific), 100 units/ml penicillin, and 100 μg/ml streptomycin (Welgene Inc., Daegu, South Korea) at 37 °C in a humidified chamber containing 95% air and 5% CO_2_. Twenty-four hours after plating, INS-1 cells were treated with 20 ng IL-1β (PeproTech, Seoul, South Korea) and 20 ng TNFα (PeproTech) for the indicated time points.

### Cell viability assay

Cells were treated with 3-(4,5-dimethylthiazol-2-yl)−2,5-diphenyl tetrazolium bromide (MTT) (Duchefa, Haarlem, Netherlands) (0.5 mg/ml) at 37 °C for 3 h. Supernatants were discarded and isopropanol was added. After incubating at 24 °C for 30 min, absorbance was measured at 570 nm using a microplate reader.

### Transmission electron microscopy (TEM) analysis

Cells (1 × 10^6^) were fixed in 4% paraformaldehyde and then in 1% osmium tetroxide. Samples were dehydrated via ethanol grade series, infiltrated with propylene oxide, and embedded with Epoxy resin (Poly bed 812 kit; Polysciences, Inc., Warrington, PA, USA). Embedded samples were cut into 65 nm-thick sections and stained with uranyl acetate and lead citrate. Samples were imaged using transmission electron microscopy (TEM, Philips CM200; Field Emission Instruments, USA), and images were acquired using XR41B CCD camera (Advanced Microscopy Techniques, MA, USA)

### Sodium carbonate extraction and sucrose density gradient fractionation of caveolae

Experiments were carried out following the detergent-free protocol developed by Song KS *et al*.^20^. Briefly, INS-1 cells were washed with ice-cold phosphate-buffered saline, trypsinised and homogenised. Sonicated cell samples were mixed with equal volumes of 80% sucrose solution in MES buffered saline (25 mM MES, pH 6.5, 0.15 M NaCl), placed in an ultracentrifuge tube, and overlaid with 4 ml of 30% sucrose and 4 ml of 5% sucrose in MES‐buffered saline containing 0.25 M Na_2_CO_3_. Gradients were generated by centrifugation at 200,000 × *g* for 18 h in a SW41 rotor (Beckman Coulter, INC., Atlanta, USA). Fractionations were collected from the top of the gradient and dissolved in 1 × Laemmli SDS sample buffer prior to western blot analysis.

### Western blotting

Cells were lysed in mammalian protein extraction buffer (GE Healthcare, Milwaukee, WI, USA). Nuclear and cytoplasmic proteins were extracted according to the NE-PER^TM^ Nuclear and Cytoplasmic Extraction Reagents manufacturer’s instructions (Thermo Fisher Scientific, Madison, WI, USA). Thirty micrograms of protein samples were separated by SDS–PAGE, transferred to nitrocellulose membranes, and incubated with specific antibodies. The following antibodies were used at the dilution indicated: anti-cav-1, anti-IL-1R1, anti-TNFR, anti-IKKα, anti-IKKβ, anti-p-IKKα/β, anti-IκB, anti-p-IκB, anti-NFκB p65, anti-p-NFκB p65 (1:1000; Cell Signalling Technology, Boston, MA, USA), and anti-β-actin (1:10,000; Santa Cruz Biotechnology, Santa Cruz, CA, USA). After washing, the membranes were incubated with a secondary antibody conjugated with horseradish peroxidase for 1 h at 24 °C. Signal was detected using Fujifilm luminescent image analyzer LAS 4000 with ECL detection kit (Millipore, Watford, UK).

### Real-time quantitative PCR

Total RNA was extracted using RNAiso Plus (Takara Bio Inc., Shiga, Japan) and was transcribed into cDNA using cDNA kit (Takara Bio Inc.). Real-time quantitative PCR was performed using the SYBR^®^ Premix Ex Taq^TM^ II, ROX plus (Takara Bio Inc.) with the following probes: rat cyclophilin (cyclo) forward 5′- GGTCTTTGGGAAGGTGAAAGAA-3′ and reverse 5′- GCCATTCCTGGACCCAAAA-3′; rat cav-1 forward 5′- GCGCACACCAAGGAGATTG-3′ and reverse 5′- CACGTCGTCGTTGAGATGCT-3′; rat IL-1β forward 5′- GCAATGGTCGGGACATAGTT -3′ and reverse 5′- AGACCTGACTTGGCAGAGGA-3′; rat iNOS forward 5′- CTCACTGTGGCTGTGGTCACCTA-3′ and reverse 5′ - GGGTCTTCGGGCTTCAGGTTA-3′. Relative levels of mRNA gene expression were calculated using the 2− ΔΔCt method.

### Annexin-V staining

The number of cells undergoing early apoptosis was determined using an Annexin-V-FITC apoptosis detection kit (BD Biosciences, Franklin Lakes, NJ, USA), according to the manufacturer’s instructions. Cells were then harvested and suspended in binding buffer, and annexin-V-FITC and Propidium Iodide (PI) were added. After incubation, stained cells (10,000 cells/sample) were analysed by flow cytometry (FACS Calibur; BD Biosciences).

### Immunofluorescence microscopy

Cell cultured on glass coverslips were fixed with 4% formaldehyde and permeabilized with PBS containing 0.5% triton X-100 and blocked with 2% BSA in PBS for 1 h. They were then incubated with mouse monoclonal antibody against cav-1, rabbit polyclonal antibody against IL-1R1 and TNFR1 overnight. After washing the coverslips were incubated with Alexa Fluor 594^TM^-conjugated anti-rabbit, DyLight^TM^ 488-conjugated anti-mouse secondary antibodies. The labelled cells were observed under a confocal microscope.

### Transfection

For small interfering RNA transfection, INS-1 cells were plated and transiently transfected with 1 pM of cav-1 siRNA or scrambled siRNA (Santa Cruz Biotechnology, Dallas, TX, USA) using Lipofectamine RNAiMAX (Invitrogen Carlsbad, CA, USA) reagent as per the manufacturer’s instructions. After 24 h, the medium was replaced with CM for various time periods. INS-1 cells were transfected with an empty vector and pcDNA3-cav-1 (Thermo Fisher Scientific) plasmid DNA using Lipofectamine 2000 reagent (Invitrogen), according to the manufacturer’s instructions.

### Animals and islet isolation

Four-week-old male C57BL/6 J mice and 6 week old male Sprague-Dawley (SD) rat were purchased from Korea Research Institute of Bioscience & Biotechnology (KRIBB, Daejeon, South Korea) and Daehan Biolink (Daehan Biolink Co. LTD., Eumsung, South Korea). All animals were kept in the animal facility for 7 d to allow for environmental adaptation. The mice were fed standard chow for 2 wk and were then sacrificed for islet isolation. The collagenase digestion technique was used as previously described^36^, and islets were separated by centrifugation on Histopaque-1077 (Sigma-Aldrich, St. Louis, MO, USA). Healthy islets of appropriate sizes were individually-picked under a stereomicroscope. The islets were dissociated into single cells by trypsinisation. All experiments were approved by the Institutional Animal Care and Use Committee of Eulji University (EUIACUC18-7) and confirmed that all experiments were performed in accordance with relevant guidelines and regulations.

### Glucose-stimulated insulin secretion assay

INS-1 cells were plated in 24-well plates and transfected with either control siRNA and cav-1 siRNA for 24 h. Cells were starved in Krebs-Ringer bicarbonate buffer without glucose for 2 h and insulin secretion was stimulated by treatment of cells with 3 mM or 17 mM glucose for 1 h. At the end of the incubation, the amount of insulin released into the supernatant was quantified using a rat insulin EIA kit (Alpco Diagnostics, Windham, NH, USA), according to the manufacturer’s instructions. The amount of insulin released was normalized to the total amount of protein.

### Statistical analysis

Results are expressed as mean ± SEM of three separate experiments. Shapiro-Wilk normality test was performed in SPSS (Statistical Analysis System Institute 2010, IBM corp., Chicago, IL, USA) and differences between the control group and the treated group were assessed via the Student’s t test. Analysis of variance (ANOVA), followed by Scheffe’s multiple comparison test, were used to determine the significance of any differences among more than two groups. P < 0.05 was considered significant.

## Supplementary information

Dataset 1
