# Supplementary material for: Upregulation of caveolin-1 and its colocalization with cytokine receptors contributes to beta cell apoptosis
Source: Sci Rep. 2019 Nov 14;9:16785. doi: 10.1038/s41598-019-53278-z (PMC6856349; doi:10.1038/s41598-019-53278-z)
Supplement: Supplementary file 1 — Dataset 1 [file 41598_2019_53278_MOESM1_ESM.docx]

**Upregulation of caveolin-1 and its colocalization with cytokine receptors contributes to beta cell apoptosis**

**Gong Deuk Bae, Eun-Young Park, Kyong Kim, Se-Eun Jang, Hee-Sook Jun and**

**Yoon Sin Oh**

**
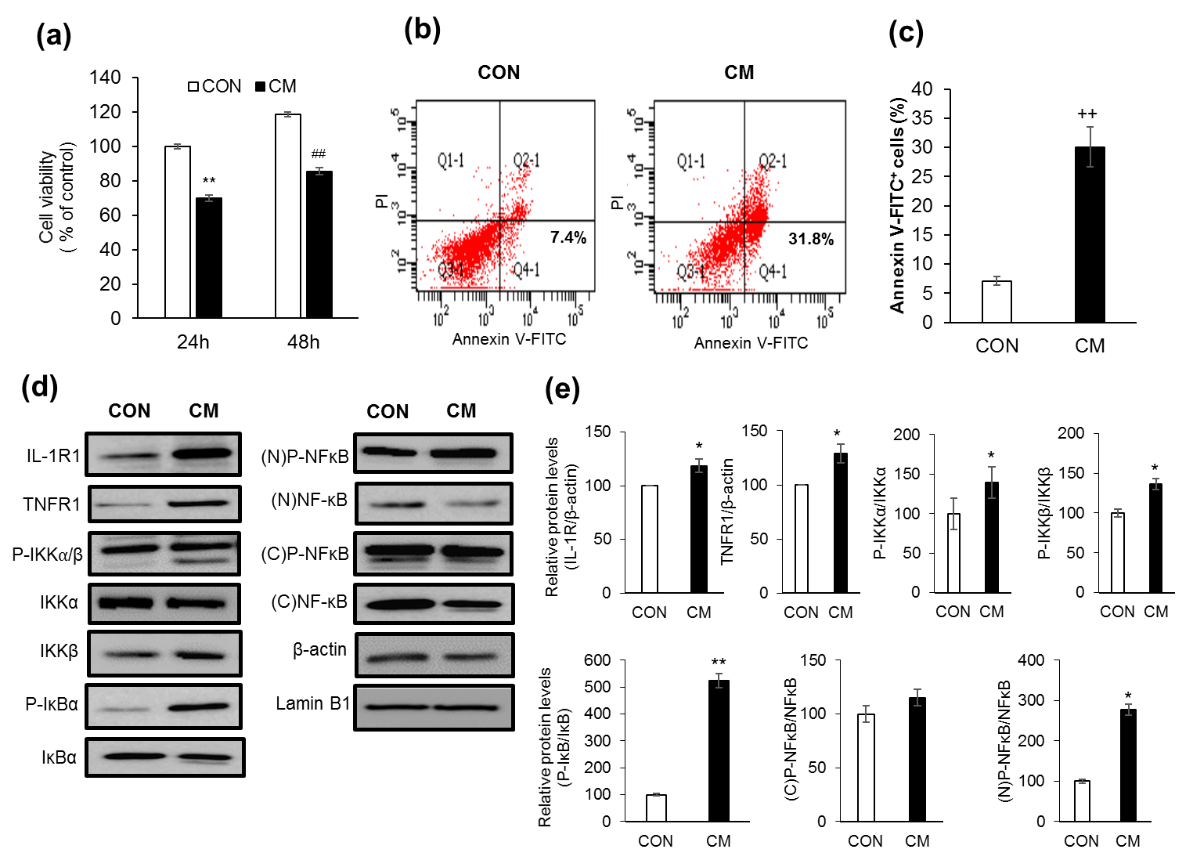
**

**Figure 1. Cytokine mixture treatment increases apoptosis via NF-κB signalling activation in INS-1 cells**

(a) INS-1 cells were incubated with or without the cytokine mixture (CM; IL-1β 20 ng/ml, TNFα 20 ng/ml) for the indicated time points, and cell viability was measured by the MTT assay. (b) Cells were treated with CM for 24 h, harvested, stained with Annexin V/propidium iodide, and apoptotic cells were evaluated by flow cytometry. (c) Quantitative data demonstrating the AnnexinV-FITC^+^ cells (%) (lower right quadrant). (d) Cells were treated with CM for 24 h and western blot analysis of cell lysates was performed with specific antibodies. Actin and Lamin B1 were used as cytosolic and nuclear loading controls, respectively. (e) The densities of western blot signals were measured, and the relative expression levels were normalized to that of actin and non-phosphorylated form (IKK, IκB, and NF-κB). Values are means ± SEM from triplicate experiments, ** p<0.005 vs. 24 h CON, ##p<0.005 vs. 48 h CON, ++p<0.005 vs. CON, *p<0.05 vs. CON.

**
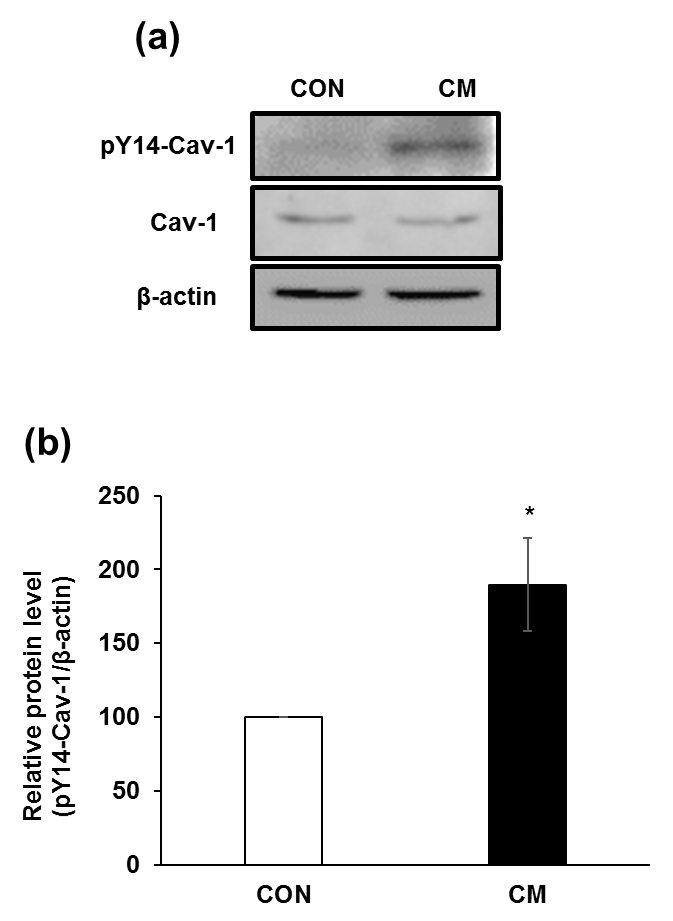
**

**Supplementary Figure 2. Cytokine mixture induced phosphorylation of cav-1 on tyrosine-14**

INS-1 cells were serum-deprived for 12 h and exposed to cytokine mixture. (a) After 2 h treatment, cells were harvested and western blot analysis of cell lysates was performed with specific antibodies. Actin was used as loading control. (b) The densities of western blot signals were measured, and the relative expression levels were normalized to that of non-phosphorylated form. Values are means ± SEM from triplicate experiments, *p<0.05 vs. CON.

**
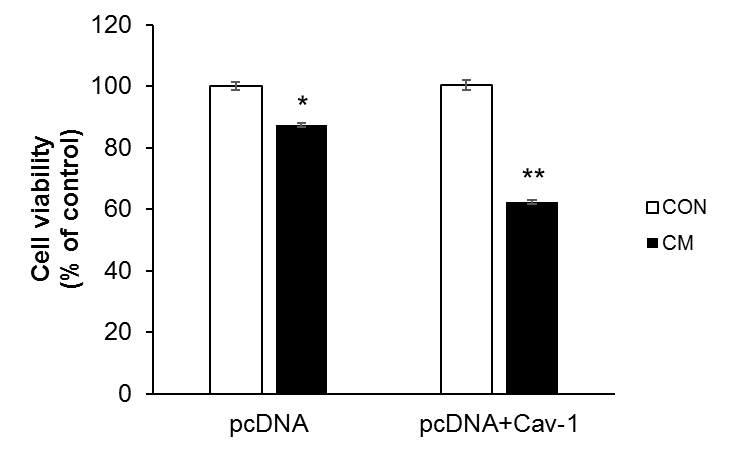
**

**Supplementary Figure 3. Overexpression of cav-1 increased cell damage induced by cytokine mixture**

INS-1 cells were transfected with pcDNA 3.1 or pcDNA3.1-Cav-1. Cell viability was measured by the MTT assay at 24 h after treatment with the cytokine mixture (CM; IL-1β 20 ng/ml, TNFα 20 ng/ml). Values are means ± SEM from triplicate experiments, *p<0.05 vs. pcDNA treated with CON, **p<0.005 vs. pcDNA treated with CM.


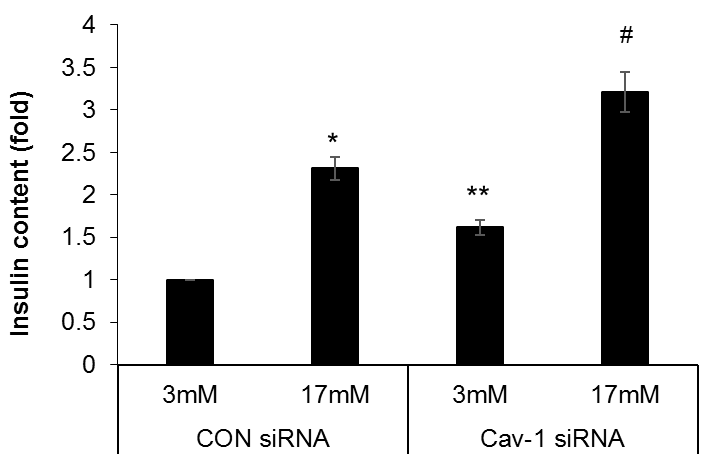


**Supplementary Figure 4. Downregulation of cav-1 expression increases glucose-stimulated insulin secretion**

CON siRNA (10 µM) or Cav-1 siRNA (10 μM) was transfected in INS-1 cells. After 24 h treatment, cells were incubated in 3 or 17 mM glucose. The amount of insulin released into the supernatant was quantified using a rat insulin EIA kit and normalized to the total protein amount. Values are means ± SEM from triplicate experiments, *p<0.05 vs. CON siRNA treated with 3 mM glucose, **p<0.005 vs. CON siRNA treated with 3 mM glucose, # p<0.05 vs. CON siRNA treated with 17 mM glucose

**Supplementary Figure 5. Uncropped scans of western blot displayed in Figure 1b and d**


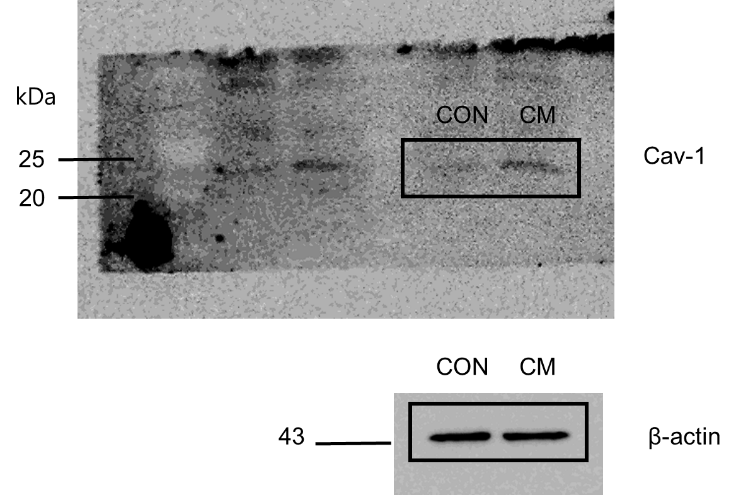


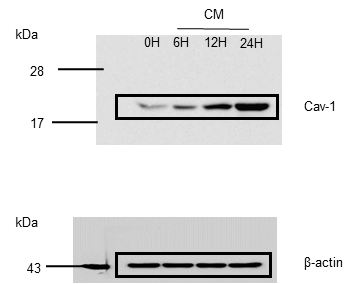


**Supplementary Figure 6. Uncropped scans of western blot displayed in Figure 2b**


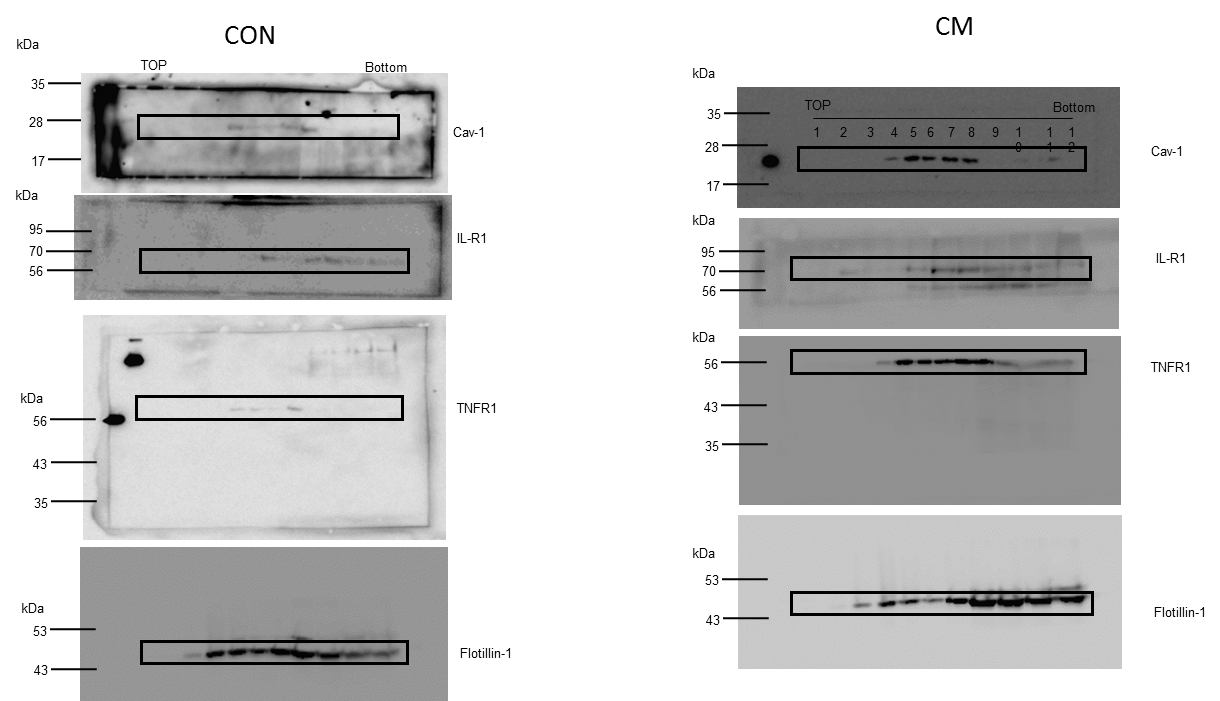


**Supplementary Figure 7. Uncropped scans of western blot displayed in Figure 3b**


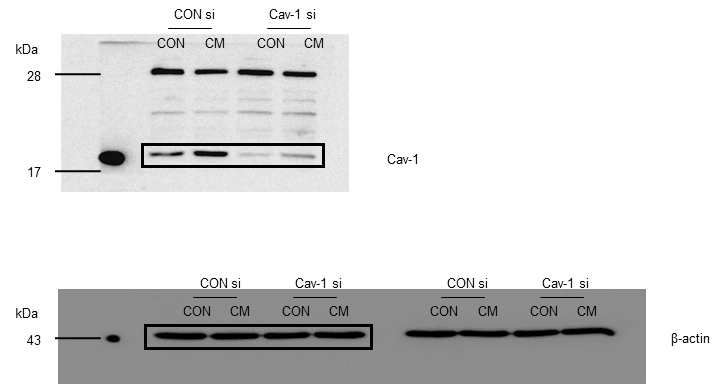


**Supplementary Figure 8. Uncropped scans of western blot displayed in Figure 5a**

**
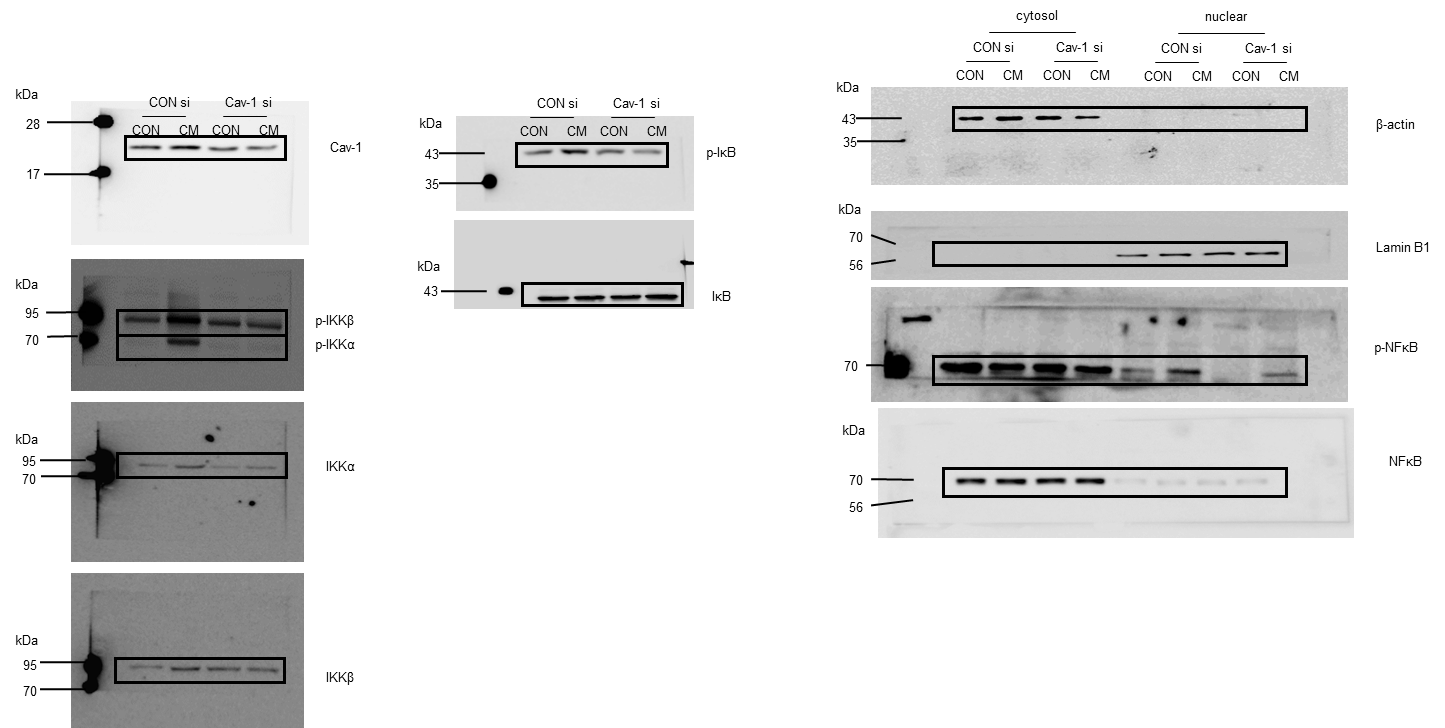
**

**Supplementary Figure 9. Uncropped scans of western blot displayed in Supplementary Figure 1d**


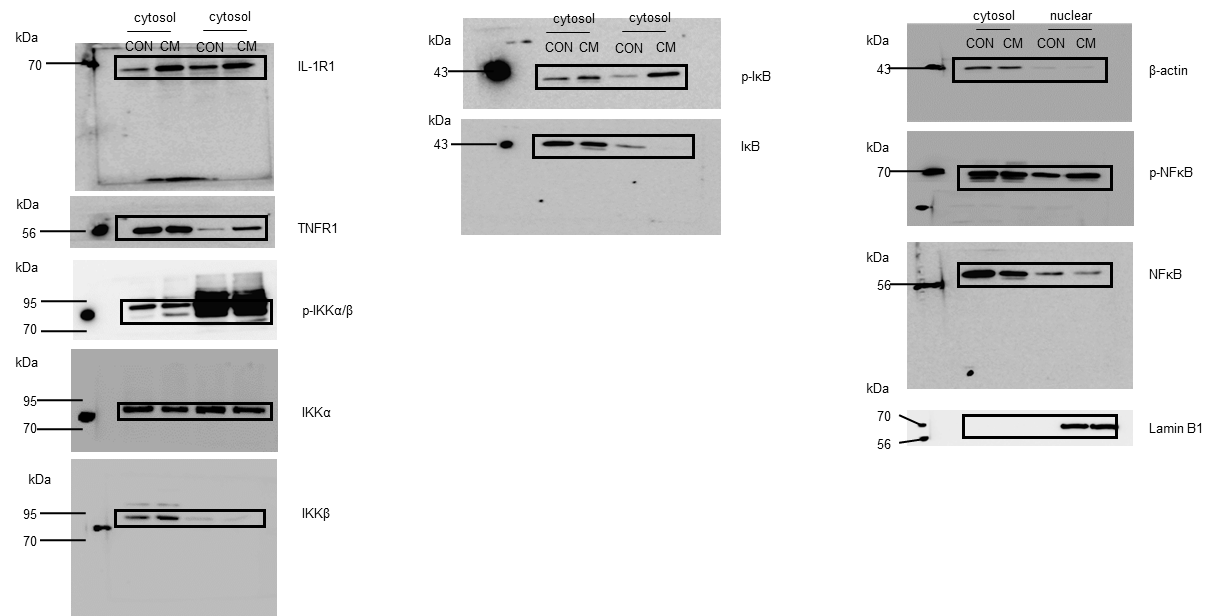


**Supplementary Figure 10. Uncropped scans of western blot displayed in Supplementary Figure 2a**

**
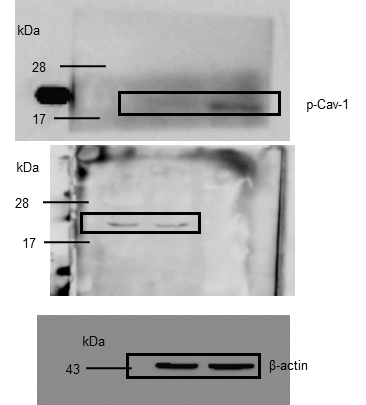
**
